# Multifractal analysis of weighted networks by a modified sandbox algorithm

**DOI:** 10.1038/srep17628

**Published:** 2015-12-04

**Authors:** Yu-Qin Song, Jin-Long Liu, Zu-Guo Yu, Bao-Gen Li

**Affiliations:** 1 Hunan Key Laboratory for Computation and Simulation in Science and Engineering and Key Laboratory of Intelligent Computing and Information Processing of Ministry of Education, Xiangtan University, Xiangtan, Hunan 411105, China; 2 College of Science, Hunan University of technology, Zhuzhou, Hunan 412007, China; 3 School of Mathematical Sciences, Queensland University of Technology, Brisbane, Q4001, Australia

## Abstract

Complex networks have attracted growing attention in many fields. As a generalization of fractal analysis, multifractal analysis (MFA) is a useful way to systematically describe the spatial heterogeneity of both theoretical and experimental fractal patterns. Some algorithms for MFA of unweighted complex networks have been proposed in the past a few years, including the sandbox (SB) algorithm recently employed by our group. In this paper, a modified SB algorithm (we call it SBw algorithm) is proposed for MFA of weighted networks. First, we use the SBw algorithm to study the multifractal property of two families of weighted fractal networks (WFNs): “Sierpinski” WFNs and “Cantor dust” WFNs. We also discuss how the fractal dimension and generalized fractal dimensions change with the edge-weights of the WFN. From the comparison between the theoretical and numerical fractal dimensions of these networks, we can find that the proposed SBw algorithm is efficient and feasible for MFA of weighted networks. Then, we apply the SBw algorithm to study multifractal properties of some real weighted networks — collaboration networks. It is found that the multifractality exists in these weighted networks, and is affected by their edge-weights.

Complex networks have attracted growing attention in many fields. More and more research works have shown that they connect with many real complex systems and can be used in various fields^1^,^2^,^3^,^4^. Fundamental properties of complex networks, such as the small-world, the scale free and communities, have been studied^5^,^6^. Song *et al.*^1^ found the self-similarity property^7^,^8^,^9^ of complex networks. Gallos *et al.* gave a review of fractality and self-similarity in complex networks^10^. At the same time, some methods for fractal analysis and how to numerically calculate the fractal dimension of complex networks have been proposed. Especially, the box-counting algorithm^11^,^12^ was generalized and applied to calculate the fractal dimension of complex networks. Subsequently, an improved algorithm was proposed to investigate the fractal scaling property in scale-free networks^13^. In addition, based on the edge-covering box counting, an algorithm was proposed to explore the self-similarity of complex cellular network^14^. A ball-covering approach and an approach defined by the scaling property of the volume were proposed to calculate the fractal dimension of complex networks^15^. Later on, box-covering algorithms for complex networks were further studied^16^,^17^.

Although fractal analysis can describe global properties of complex networks, it is inadequate to describe the complexity of complex networks by a single fractal dimension. For systematically characterizing the spatial heterogeneity of a fractal object, multifractal analysis (MFA) has been introduced^18^,^19^. MFA has been widely applied in many fields, such as financial modeling^20^,^21^, biological systems^22^,^23^,^24^,^25^,^26^,^27^,^28^,^29^,^30^,^31^,^32^, geophysical systems^33^,^34^,^35^,^36^,^37^,^38^,^39^,^40^ and also complex networks^41^,^42^,^43^,^44^,^45^. Lee *et al.*^46^ mentioned that MFA is the best tool to describe the probability distribution of the clustering coefficient of a complex network. Some algorithms were proposed for MFA of unweighted complex networks in past a few years^41^,^42^,^43^,^44^,^45^. Furuya and Yakubo^41^ pointed out that a single fractal dimension is not enough to characterize the fractal property of a scale-free network when the network has a multifractal structure. They also introduced a compact-box-burning (CBB) algorithm for MFA of complex networks. Wang *et al.*^42^ proposed an improved fixed-size box-counting algorithm to study the multifractal behavior of complex networks. Then this algorithm was improved further by Li *et al.*^43^. They applied the improved fixed-size box-counting algorithm to study multifractal properties of a family of fractal networks proposed by Gallos *et al.*^47^. Recently, Liu *et al.*^45^ employed the sandbox (SB) algorithm proposed by Tél *et al.*^48^ for MFA of complex networks. The comparison between theoretical and numerical results of some networks showed that the SB algorithm is the most effective and feasible algorithm to study the multifractal behavior of unweighted networks^45^.

However, all the algorithms for MFA in refs 41, 42, 43, 44, 45 are just feasible for unweighted networks. Actually, there are many weighted networks in real world^49^,^50^,^51^, but few works have been done to study the fractal and multifractal properties of the weighted networks. Recently, an improved box-covering algorithm for weighted networks was proposed by Wei *et al.*^52^. They applied the box-covering algorithm for weighted networks (BCANw) to calculate the fractal dimension of the “Sierpinski” weighted fractal network (WFN)^53^ and some real weighted networks. But the BCANw algorithm was only designed for calculating the fractal dimension of weighted networks.

In this work, motivated by the idea of BCANw, we propose a modified sandbox algorithm (we call it SBw algorithm) for MFA of weighted networks. First, we use the SBw algorithm to study the multifractal property of two families of weighted fractal networks (WFNs): “Sierpinski” WFNs and “Cantor dust” WFNs introduced by Carletti *et al.*^53^. We also discuss how the fractal dimension and generalized fractal dimensions change with the edge-weights of the WFN. Through the comparison between the theoretical and numerical fractal dimensions of these networks, we check whether the proposed SBw algorithm is efficient and feasible for MFA of weighted networks. Then, we apply the SBw algorithm to study multifractal properties of some real weighted networks — collaboration networks^54^.

## Results and Discussion

### Multifractal properties of two families of weighted fractal networks

In order to show that the SBw algorithm for MFA of weighted network is effective and feasible, we apply our method to study the multifractal behavior of the “Sierpinski” WFNs and the “Cantor dust” WFNs^53^. These WFNs are constructed by Iterated Function Systems (IFS)^55^, whose Hausdorff dimension is completely characterized by two main parameters: the number of copies *s* > 1 and the scaling factor 0 < *f* < 1 of the IFS. In this case, the fractal dimension of the fractal weighted network is called the similarity dimension and given by^53^:


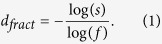


**Figure 1:**
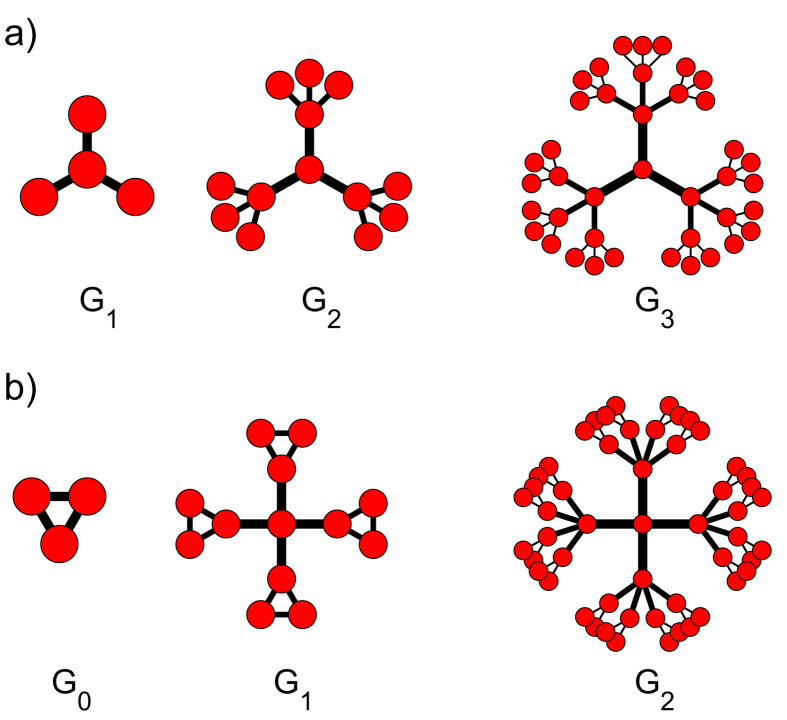
(**a**) The “Sierpinski” weighted fractal networks, s = 3, f = 1/2 and *G*_0_ is composed by a single node. From the left to the right, the 1th generation *G*_1_, the 2th generation *G*_2_, and the 3th generation *G*_3_ are shown. The fractal dimension of the limit network is log(3)/log(2) ≈ 1.5850. (**b**) The “Cantor dust” weighted fractal networks, s = 4, f = 1/5 and *G*_0_ is a triangle. From the left to the right, the 0th generation *G*_0_, the 1th generation *G*_1_, and the 2th generation *G*_2_ are shown. The fractal dimension of the limit network is log(4)/log(5) ≈ 0.8614.

To construct “Sierpinski” WFNs and “Cantor dust” WFNs^53^, a single node and a triangle is set as a initial network *G*_0_ respectively. The first a few steps to construct them are shown in parts a) and b) of Fig. 1 respectively.

We first consider two “Sierpinski” WFNs with parameters *s* = 3, *f* = 1/2 and *s* = 3, *f* = 1/3 respectively. Considering the limitation of the computing capability of our computer, we construct the 8th generation *G*_8_ of these two networks. There are 9841 nodes and 9837 edges in the *G*_8_ of these two networks. For the case *s* = 3, *f* = 1/2, the edge-weights of *G*_8_ are equal to 1, 1/2, 1/4, 1/8, 1/16, 1/32, 1/64, 1/128, respectively; the diameter of *G*_8_ is less than 4. When we use the SBw algorithm for MFA of *G*_8_, radiuses *r* of sandboxes are set to 1/128, 1/128 + 1/64, ···, 1 + 1/2 + 1/4 + 1/8 + 1/16 + 1/32 + 1/64 + 1/128, respectively for this case. We can do similar analysis for *G*_8_ of network with *s* = 3, *f* = 1/3. It is an important step to look for an appropriate range of *r* (*i*.*e*., *r* ∈ [*r*_*min*_, *r*_*max*_]) for obtaining the generalized fractal dimensions *D*(*q*) (defined by equations (6) and (7)) and the mass exponents *τ*(*q*) (defined by equation (5)). In this paper, we set the range of *q* values from −10 to 10 with a step of 1.

**Figure 2:**
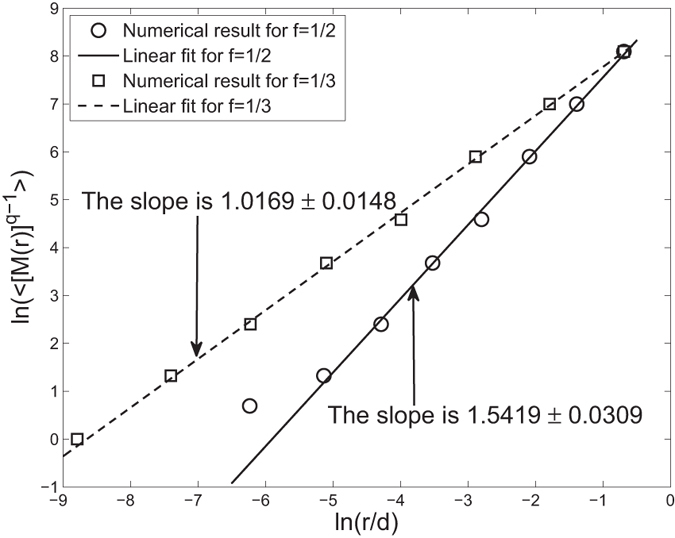
Examples of fractal analysis of the “Sierpinski” weighted fractal networks *G*_8_ with 9841 nodes. Here, copy factor *s* = 3 and the scaling factor *f* = 1/2, 1/3, respectively. By means of the least square fit, the slope of the reference lines are 1.5419 ± 0.0309 and 1.0169 ± 0.0148 respectively. The theoretical result is 1.5850 (for *f* = 1/2) and 1.0 (for *f* = 1/3), respectively.

When *q* = 0, *D*(0) is the fractal dimension of a complex network. Now we adopt the SBw algorithm to estimate the fractal dimension of two “Sierpinski” WFNs with parameters *s* = 3, *f* = 1/2 and *s* = 3, *f* = 1/3 respectively. We show the linear regression of ln(〈[*M*(*r*)]^*q*−1^〉) against (*q* − 1)ln(*r*/*d*) for *q* = 0 in Fig. 2. By means of the least square fit, the slope of the reference lines are estimated to be 1.5419 and 1.0169, with standard deviations 0.0309 and 0.0148, respectively. It means that the numerical fractal dimension is 1.5419 ± 0.0309 and 1.0169 ± 0.0148, respectively; they are very close to the theoretical similarity dimension 1.5850 and 1.0 respectively. Hence we can say that the numerical fractal dimension obtained by the SBw algorithm is very close to the theoretical similarity dimension for a “Sierpinski” WFN.

To further check the validity of the SBw algorithm, let the copy factor *s* be 3 and the scaling factor *f* be any positive real number in the range 0 < *f* < 1. From Equation (1), we can get the relationship between the fractal dimension and the scaling factor *f* of the “Sierpinski” WFN as:


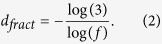


**Figure 3:**
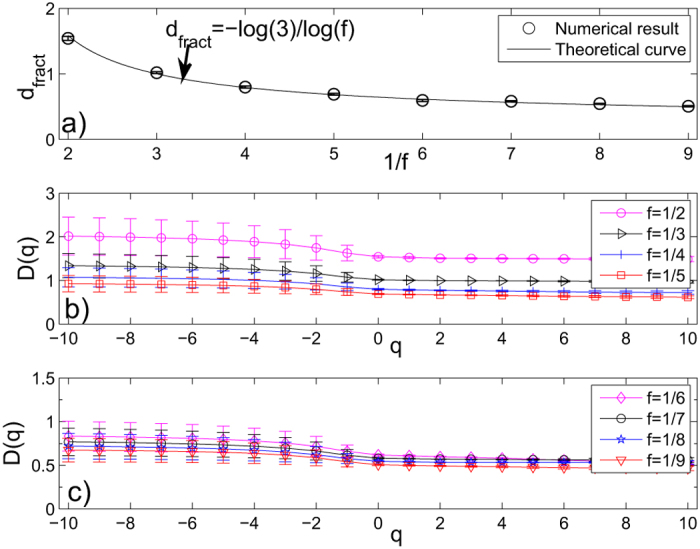
(**a**) The fractal dimensions and their standard deviations of *G*_8_ of “Sierpinski” WFNs with parameter *s* = 3. The solid curve represents the theoretical *d*_*fract*_ given by Eq. (2), circles are the numerical fractal dimensions estimated by the SBw algorithm. (**b**,**c**) The generalized fractal dimensions *D*(*q*) curves and their standard deviations of the 8th generation *G*_8_ of “Sierpinski” WFNs estimated by the SBw algorithm. Here, the parameter *s* = 3, *f* = 1/2, 1/3, 1/4, 1/5 and 1/6, 1/7, 1/8, 1/9, respectively. Each error bar takes twice length to the standard deviation for all the results.

For each value of *f* = 1/2, 1/3, 1/4, 1/5, 1/6, 1/7, 1/8, 1/9, we calculate fractal dimensions and their standard deviations of the 8th generation “Sierpinski” WFN *G*_8_ by the SBw algorithm. The results are shown in part a) of Fig. 3, where each error bar takes twice length to the standard deviation. This figure shows that the numerical fractal dimensions obtained by the SBw algorithm agree well with the theoretical fractal dimensions of these networks. This figure also shows that the fractal dimension of WFNs is affected by the edge-weight. This result coincides with the conclusion obtained by Wei *et al.*^52^.

Hence we can apply the SBw algorithm to calculate the generalized fractal dimensions *D*(*q*) and their standard deviations of “Sierpinski” WFNs. In parts b) and c) of Fig. 3, we show the generalized fractal dimensions *D*(*q*) of the 8th generation *G*_8_ of “Sierpinski” WFNs, with the parameter *s* = 3, *f* = 1/2, 1/3, 1/4, 1/5 and 1/6, 1/7, 1/8, 1/9 respectively. From these figures, we can see that all the 8th generation *G*_8_ of “Sierpinski” WFNs for different *f* have multifractal property, and the multifractal property of these weighted networks is affected by their edge-weights. The result also shows that the generalized fractal dimension *D*(*q*) almost decreases with the decrease of the scaling factor *f* for any *q*.

**Figure 4:**
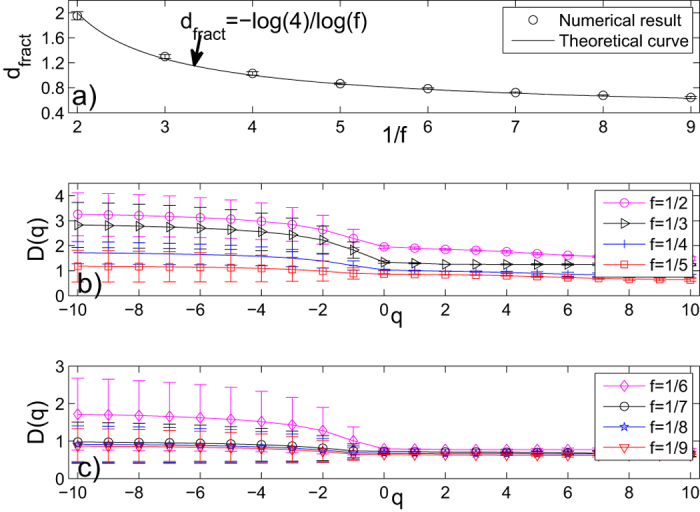
(**a**) The fractal dimensions and their standard deviations of *G*_5_ of “Cantor dust” WFNs with parameter *s* = 4. The solid curve represent the theoretical *d*_*fract*_ given by Eq. (1), circles indicate the numerical fractal dimension estimated by the SBw algorithm. (**b**,**c**) The generalized fractal dimensions *D*(*q*) curves and their standard deviations of *G*_5_ of “Cantor dust” WFNs estimated by the SBw algorithm. Here, the parameter *s* = 4, *f* = 1/2, 1/3, 1/4, 1/5 and 1/6, 1/7, 1/8, 1/9, respectively. Each error bar takes twice length to the standard deviation for all the results.

For “Cantor dust” WFNs, we can only construct the 5th generation networks with *s* = 4 and *f* = 1/2, 1/3, 1/4, 1/5, 1/6, 1/7, 1/8, 1/9, respectively. We first calculate fractal dimensions and their standard deviations of these WFNs by the SBw algorithm. The results are shown in part a) of Fig. 4. From this figure, we can see that the numerical fractal dimensions obtained by the SBw algorithm are very close to the theoretical fractal dimensions *d*_*fract*_ = −log(4)/log(*f*) for these WFNs. Then we apply the SBw algorithm to calculate the generalized fractal dimensions *D*(*q*) and their standard deviations of these “Cantor dust” WFNs. We show the numerical results of the 5th generation *G*_5_ of “Cantor dust” WFNs in parts b) and c) of Fig. 4. From these figures, we can see that all *D*(*q*) curves are nonlinear. It indicates that all these weighted networks have multifractal property. Similar to “Sierpinski” WFNs, the multifractal property of these networks is affected by their edge-weights.

The multifractal property of “Sierpinski” WFNs and “Cantor dust” WFNs revealed by the SBw algorithm indicates that these model networks are very complicated, and cannot be characterized by a single fractal dimension.

### Applications: multifractal properties of three collaboration networks

Now we apply the SBw algorithm to study multifractal properties of some real networks. We study three collaboration networks: the high-energy theory collaboration network^54^, the astrophysics collaboration network^54^, and the computational geometry collaboration network^56^.

#### High-energy theory collaboration network

**Figure 5:**
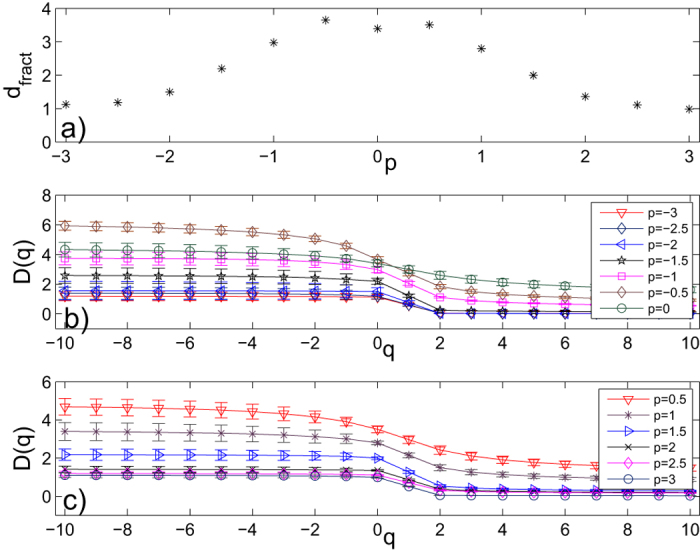
(**a**) The relation between values of the fractal dimension of the High-energy theory collaboration networks and values of *p*. We set the range of the *p* values from −3 to 3 with a step of 0.5. (**b**,**c**) The generalized fractal dimensions *D*(*q*) curves and their standard deviations of the the High-energy theory collaboration network by using the SBw algorithm. Here, the range of the *p* values from −3 to 3 with a step of 0.5. Each error bar takes twice length to the standard deviation for all the results.

This network has 8361 nodes and 15751 edges, the edge-weights are defined as^54^:


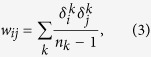


where *n*_*k*_ is the number of co-author in the *k*th paper (excluding single authored papers), 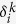 equals to 1 if the *i*th scientist is one of the co-author of the *k*th paper, otherwise it equals to 0. The data contains all components of the network, for a total of 8361 scientists, not just the largest component of 5835 scientists. When two authors share many papers, the weight value is larger, thus the distance is less. So, in Equation(9), *p* had better be a negative number (e.g. −1 given by Newman^54^). For different values of *p*, we can calculate the shortest path by Equation(9) and obtain different weighted networks. Then we apply the SBw algorithm to calculate the generalized fractal dimensions *D*(*q*) and their standard deviations of the largest component of the network with 5835 nodes. We show the relation between the numerical fractal dimension of the High-energy theory collaboration networks and values of *p* in part a) of Fig. 5. From this figure, we can see the value of fractal dimension decreases with the increase of the absolute value of *p*, the values of fractal dimensions are almost symmetric about the vertical axis. We show the numerical results on the generalized fractal dimensions *D*(*q*) of the High-energy theory collaboration networks for different values of *p* in parts b) and c) of Fig. 5. From these figures, we can see that all the High-energy theory collaboration networks for different *p* have multifractal property, and the multifractal property of these weighted networks is affected by the edge-weight. We can also see that the generalized fractal dimensions *D*(*q*) almost decrease with the increase of the absolute value of *p*.

#### Astrophysics collaboration network

**Figure 6:**
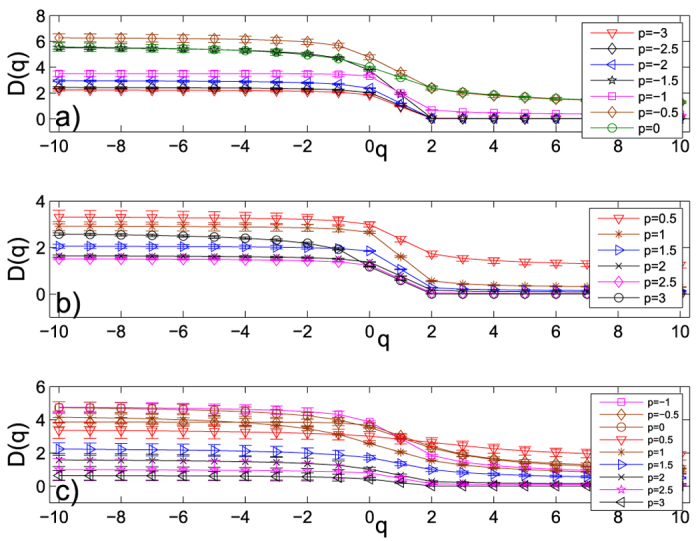
The generalized fractal dimensions *D*(*q*) curves and their standard deviations of (**a,b**) the astrophysics collaboration networks, and (**c**) the computational geometry collaboration networks estimated by the SBw algorithm. Here, we set the range of the *p* values from −1 to 3 with a step of 0.5. Each error bar takes twice length to the standard deviation for all the results.

This network has 16706 nodes and 121251 edges, the edge-wights is defined as Equation(3). Here, the data contains all components of the network, for a total of 16706 scientists, not just the largest component of 14845 scientists. When two authors share many papers, the weight value is larger, thus the distance is less. So, in Equation(9), *p* had also better be a negative number (e.g. −1 given by Newman^54^). We calculate the shortest path by Equation (9) and obtain some weighted networks with different values of *p*. Then we apply the SBw algorithm to calculate the generalized fractal dimensions *D*(*q*) and their standard deviations of the largest component of the network with 14845 nodes. We show the numerical results of the astrophysics collaboration networks in parts a) and b) of Fig. 6. From this figure, we can see that these networks also have multifractal property, and the multifractal property of these weighted networks is affected by the edge-weight.

#### Computational geometry collaboration network

The authors collaboration network in computational geometry was produced from the BibTeX bibliography which obtained from the Computational Geometry Database. This network has 7343 nodes and 11898 edges. Two authors are linked with an edge, if and only if they wrote a common paper or book, etc. The value of edge-weight is the number of common works, so the value is one integer, such as 1, 2, 3, ···, etc. The data contains all components of the network, for a total of 7343 scientists, not just the largest component of 3621 scientists. The data can be got from Pajek Data^56^. When two authors share many papers, the weight value is larger, thus the distance is less. So, in Equation (9), *p* had better be a negative number. We calculate the shortest path by Equation (9) and obtain some weighted networks with different values of *p*. Then we apply the SBw algorithm to calculate the generalized fractal dimensions *D*(*q*) and their standard deviations of the largest component of the network with 3621 nodes. Because the way to define the weight of this network is different from another two real networks, we can only calculate the generalized fractal dimensions *D*(*q*) and their standard deviations of the largest component of the network with 3621 nodes for *p* ≥ −1. We show the numerical results of the computational geometry collaboration networks in part c) of Fig. 6. From this figure, we can also see that these networks have multifractal property, and the multifractal property of these weighted networks is affected by the edge-weight (but the impact is relatively small).

## Conclusions

In this paper, a modified sandbox algorithm (we call it SBw algorithm) for MFA of weighted networks is proposed. First, we used the SBw algorithm to study the multifractal property of two families of weighted fractal networks (WFNs): “Sierpinski” WFNs and “Cantor dust” WFNs. We also discussed how the fractal dimension and generalized fractal dimensions change with the edge-weights of the WFN. From the comparison between the theoretical and numerical fractal dimensions of these networks, we can find that the proposed SBw algorithm is efficient and feasible for MFA of weighted networks.

In addition, we applied the SBw algorithm to study the multifractal properties of some real networks — the high-energy theory collaboration network, the astrophysics collaboration network, and the computational geometry collaboration network. We found that multifractality exists in these weighted networks, and is also affected by their edge-weight. Our result indicates that multifractal property of weighted networks are affected both by their edge weight and their topology structure.

## Methods

### Multifractal analysis

The fixed-size box-counting algorithm is one of the most common and effective algorithms to explore multifractal properties of fractal sets^19^. For a support set *E* in a metric space Ω and a normalized measure *μ* (i.e. 0 ≤ *μ*(Ω) ≤ 1), we consider the partition sum:


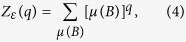


where *q* ∈ *R*, and the sum runs over all different non-overlapping boxes *B* which cover the support set *E* with a given size *ε*. The mass exponents *τ*(*q*) of the measure *μ* is defined as:


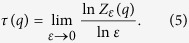


The generalized fractal dimension *D*(*q*) of the measure *μ* is defined as:


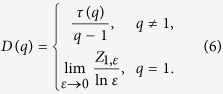


where *Z*_1,*ε*_ = ∑_*μ*(*b*)≠0_*μ*(*B*)ln*μ*(*B*). A numerical estimation of the generalized fractal dimension *D*(*q*) can be got from the linear regression of ln*Z*_*ε*_(*q*)/*q* − 1 against ln*ε* for *q* ≠ 1, *Z*_1,*ε*_ against ln*ε* for *q* = 1, respectively.

Tèl *et al.*^48^ proposed the sandbox (SB) algorithm for MFA of fractal sets which is an extension of the box-counting algorithm^19^. The generalized fractal dimensions *D*(*q*) are defined as^48^:


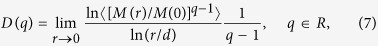


where *M*(*r*) is the number of points in the sandbox with radius *r*, *M*(0) is the number of all points in the fractal object. It is denoted the brackets 〈⋅〉 to take statistical average over randomly chosen centers of the sandboxes. From Equation (7) we can get the relation:


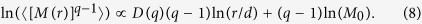


From Equation (8), we can obtain an estimation of the generalized fractal dimension *D*(*q*) by the linear regression of ln(〈[*M*(*r*)]^*q*−1^〉) against (*q* − 1)ln(*r*/*d*). Then, we can also get the mass exponents *τ*(*q*) through *τ*(*q*) = (*q* − 1)*D*(*q*). Specifically, *D*(0) is the fractal dimension, *D*(1) is the information dimension, *D*(2) is the correlation dimension of the fractal object, respectively.

### A modified sandbox algorithm for multifractal analysis of weighted networks

Recently, our group employed the SB algorithm proposed by Tél *et al.*^48^ for MFA of unweighted complex networks^45^. In the SB algorithm^45^, the radiuses *r* of the sandbox are set to be integers in the range from 1 to the diameter of the unweighted network. However, in weighted networks, the values of edge-weights could be any real numbers excluding zero and the shortest path is defined by the path between two nodes such that the sum of values of its edge-weights to be minimized in some way^57^. So, the shortest path between two nodes could be any real numbers excluding zero. In this paper, for weighted networks, we denote the length of shortest path between node *i* and node *j* by *d*_*ij*_, and *d*_*ij*_ is defined as^52^:


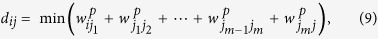


where *w*_*kh*_ means the edge-weight of directly connecting node *k* and node *h* in a path, *j*_*m*_(*m* = 1, 2, ···) are IDs of nodes and *p* is a real number. In particular, when *p* equal to zero, the length of the shortest path given by Equation(9) is the same as unweighted networks^57^. If the edge-weight is only a number without obvious physical meaning, we set *p* equals to 1, such as the “Sierpinski” WFN^53^. In some real weighted networks, one case is that the bigger edge-weight of between any two nodes is, the less distance is, such as the collaboration networks, where *p* < 0^54^; the other case is that the bigger edge-weight of between any two nodes is, the further distance is, such as the real city network and the “Sierpinski” WFN, where *p* > 0.

The SB algorithm is unfeasible for MFA of weighted networks because we cannot obtain enough numbers of boxes (even only one sandbox we can obtain when the diameter of the weighted network is less than one). Wei *et al.*^52^ proposed an improved box-covering algorithm for fractal analysis of weighted network (BCANw). In the present work, motivated by the idea of BCANw, we propose a modified sandbox algorithm (we call it SBw algorithm) for MFA of weighted networks. The SBw algorithm can deal with the multifractal property (hence can also deal with the fractal property) of weighted networks.

Before we apply the SBw algorithm for MFA of weighted networks, we need to calculate the shortest-path distance matrix *D* of the network and set the range of radiuses *r* of the sandboxes. The detail is given as:

- A network is mapped to an adjacent matrix *W*_*N* × *N*_, where *N* is the total number of nodes in the network. For any given real numbers *p*, the elements of the adjacent matrix 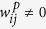 is the edge-weight between directly connecting nodes *i* and *j*, otherwise 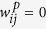. According to the adjacent matrix *W*_*N* × *N*_, we can calculate the shortest path distance matrix *D* by applying the Floyd’s algorithm^58^ of Matlab BGL toolbox^59^;
- For any given real numbers *p*, order the edge-weights 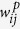 as *w*_1_ ≤ *w*_2_ ≤ ··· ≤ *w*_*m*_, where *m* is the number of edge-weights. From the fractal theory, we should look for an appropriate range of radiuses *r* to perform the least square linear fit and then obtain the generalized fractal dimensions *D*(*q*) accurately. We tried choosing the radius *r* from 0 to diameter *d* with equal (linearly or logarithmically) intervals. But we found it is hard to look for an appropriate range of radiuses *r* to perform the least square linear fit and then obtain the generalized fractal dimensions *D*(*q*) of weighted complex networks we considered accurately. So the radiuses *r* of the sandboxes are obtained by accumulating the value of the edge-weights until it is larger than the diameter *d* of the network. So, we can get the set of radiuses (denoted as *R*), where 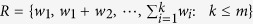 and 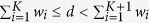. Specifically, for any *i*, *j*, if *w*_*i*_ = *w*_*j*_ = 1, then the radius set *R* is the same as the SB algorithm for unweighted network.

In this sense, the SBw algorithm can be applied to calculate the mass exponents *τ*(*q*) and the generalized fractal dimensions *D*(*q*) not only for unweighted network but also for weighted networks. Now we propose a modified SB algorithm (SBw) for MFA of weighted network as:

- Initially, ensure that all nodes in the network are not covered and not selected as a center of a sandbox.
- Set every element in the radius set *R* as the radius *r* of the sandbox which will be used to cover the nodes, where *R* is obtained as above. (in the SB algorithm the radius *r* in the range *r* ∈ [1, *d*], where *d* is the diameter of the network).
- Rearrange the nodes of the entire network into a random order. Make sure the nodes of the network are randomly chosen as the center of a sandbox.
- According to the size *N* of networks, choose the first 1000 nodes in a random order as the center of 1000 sandboxes, then for each sandbox, search all the neighbor nodes which have a distance to the center node within *r*.
- Count the number of nodes in each sandbox of radius *r*, denote the number of nodes in each sandbox of radius *r* as *M*(*r*).
- Calculate the statistical average 〈[*M*(*r*)]^*q*−1^〉 of [*M*(*r*)]^*q*−1^ over all 1000 sandboxes of radius *r*.
- For different values in the radius set *R*, repeat steps (2) to (6) to obtain the statistical average 〈[*M*(*r*)]^*q*−1^〉 and then use 〈[*M*(*r*)]^*q*−1^〉 for linear regression.

## Additional Information

**How to cite this article**: Song, Y.-Q. *et al.* Multifractal analysis of weighted networks by a modified sandbox algorithm. *Sci. Rep.* **5**, 17628; doi: 10.1038/srep17628 (2015).
